# Antibiotic prophylaxis in pregnant with premature rupture of ovular membranes: systematic review and meta-analysis

**DOI:** 10.31744/einstein_journal/2022RW0015

**Published:** 2022-11-18

**Authors:** Ana Maria Gomes Pereira, Gabriel Duque Pannain, Bruna Helena Gonçalez Esteves, Maria Luiza de Lima Bacci, Maria Luiza Toledo Leite Ferreira da Rocha, Reginaldo Guedes Coelho Lopes

**Affiliations:** 1 Instituto de Assistência Médica ao Servidor Público Estadual de São Paulo São Paulo SP Brazil Instituto de Assistência Médica ao Servidor Público Estadual de São Paulo, São Paulo, SP, Brazil.

**Keywords:** Fetal membranes, premature rupture, Gestational age, Pregnant women, Antibiotic prophylaxis, Anti-bacterial agents, Infant, premature

## Abstract

**Objective:**

To perform a systematic review and meta-analysis of randomized clinical trials that compared the use of antibiotics *versus* placebo in premature rupture of membranes preterm and evaluated maternal, fetal and neonatal outcomes in pregnant women with premature rupture of ovular membranes at a gestational age between 24 and 37 weeks.

**Methods:**

A search was conducted using keywords in PubMed, Cochrane, *Biblioteca Virtual em Saúde* and *Biblioteca Digital de Teses e Dissertações da USP* between August 2018 and December 2021. A total of 926 articles were found. Those included were randomized clinical trials that compared the use of antibiotics *versus* placebo in the premature rupture of preterm membranes. Articles referring to antibiotics only for *streptococcus agalactiae* prophylaxis were excluded. The retrieved articles were independently and blindly analyzed by two reviewers. A total of 24 manuscripts met the inclusion criteria and 21 articles were included for quantitative analysis.

**Results:**

Among the maternal outcomes analyzed, there was a prolongation of the latency period that was ≥7 days. In addition, we observed a reduction in chorioamnionitis in the group of pregnant women who used antibiotics. As for endometritis and other maternal outcomes, there was no statistically significant difference between the groups. Regarding fetal outcomes, antibiotic prophylaxis worked as a protective factor for neonatal sepsis. Necrotizing enterocolitis and respiratory distress syndrome showed no statistically significant differences.

**Conclusion:**

The study showed positive results in relation to antibiotic prophylaxis to prolong the latency period, new randomized clinical trials are needed to ensure its beneficial effect.

**Prospero database registration:**

(www.crd.york.ac.uk/prospero) under number CRD42020155315.

## INTRODUCTION

Premature rupture of the ovular membranes (PRPM) is defined as a spontaneous rupture of the chorionic and amniotic membranes that often occurs before the onset of labor, regardless of gestational age.^(1)^ When this rupture occurs before 37 weeks, it is called preterm PRPM, and it is an important cause of perinatal morbidity and mortality. The time elapsed between the rupture and the spontaneous onset of labor is defined as the latency period, and its duration is directly correlated with the risk of maternal infection and inversely with gestational age.^(2)^

Premature rupture of the ovular membranes occurs from 8% to 10% of pregnancies and up to 40% of preterm births result from preterm PRPM. Such number accounts for 20% of perinatal deaths. A recent Brazilian study in a referral maternity hospitals showed that 29% of preterm births were due to preterm PRPM and they occurred in 3.5% of a total of 33,740 deliveries.^(3)^ There are some risk factors described for the occurrence of PRPM. Among the modifiable factors, those that stand out are cervicovaginitis, isthmocervical incompetence, smoking, amniocentesis, chorionic villus sampling, intercourse, vitamin C and mineral deficiency, as well as repeated cervical examinations.^(3)^

However, the non-modifiable risk factors are history of previous surgeries, history of PRPM, vaginal bleeding, placenta previa, placental abruption, marginal insertion of the umbilical cord, and uterine hyperdistension (multiple pregnancy and polyhydramnios).^(4)^ The infectious process seems to be one of the most important one and this seems to lead to an inflammatory reaction, which alters the tissue structure of the membrane, weakening it and, thus, allowing its rupture.^(1)^ The main agents involved in this pathophysiology are *Gardnerella vaginalis, Neisseria* gonorrhoeae, Streptococcus agalactiae, Escherichia coli, and *Bacteroides sp.*^(3)^

The diagnosis of PRPM can be extremely easy, when the anamnesis and physical examination are enough to clarify it, or extremely difficult, when not even the most advanced complementary exams are convinced of the rupture. Fortunately, anamnesis and physical examination establish the diagnosis in 90% of cases.^(4)^ Premature rupture of the ovular membranes is associated with important maternal and perinatal complications, and it involves more injuries when occurring far from term.

Among maternal complications, chorioamnionitis, endometritis and bacteremia are the most frequent. Maternal sepsis is rare when there is adequate obstetric care in face of maternal signs of infection.^(5,6)^ The frequency and severity of neonatal complications vary based on gestational age. Respiratory distress syndrome (RDS) is the most common complication at any gestational age as well as other morbidities, including: necrotizing enterocolitis (NEC), peri-intraventricular hemorrhage (IVH) and sepsis.^(2,4)^

Premature rupture of the ovular membranes in women with a gestational age of less than 37 weeks remains a frequent problem in obstetric practice and there are several controversies regarding the medical management to be taken. Among the main points of disagreement are the indication of expectant management based on its diagnosis, the need for hospitalization, the use of tocolysis, and corticosteroids. In addition, there are the methods used to diagnose infection, the ideal time of delivery, and the use of antibiotics both for prophylaxis of infection by Group B *streptococcus*, as well as to increase the latency period.^(7,8)^

Therefore, scientific research related to PRPM including the analysis of different behaviors and maternal and perinatal outcomes are of great importance for the updating and possible standardization of routines in services that is searching for benefits against the morbidity and mortality associated with its occurrence.

The aim of this study was to perform a systematic review and meta-analysis of randomized clinical trials that compared the use of antibiotic prophylaxis to increase the latency period, and evaluated the maternal, fetal and neonatal outcomes of pregnant women with PRPM at gestational age between 24 and 37 weeks.

## METHODS

### Protocol registration

Criteria used for the search were the ones recommended by the PRISMA (Preferred Reporting Items for Systematic Reviews and Meta-Analyses). The guidelines established by the AMSTAR 2 (A Measurement Tool to Assess Systematic Reviews) tool were also followed to check whether the systematic review was done properly.

### Electronic search and search strategy

The following keywords were used: “antibiotic”, “antibiotics”, “anti-bacterial”, “antimicrobial”, “antibiotic prophylaxis”, “antibiotics and fetal membranes”, “premature rupture of membranes”, “premature rupture of membranes”, “fetal membranes”, “preterm premature rupture of fetal membranes”, “fetal membranes”, and “PRPM”.

Searches were carried out in PubMed, Cochrane, *Biblioteca Virtual em Saúde* and *Biblioteca Digital de Teses e Dissertações da USP* on August 22, 2018. There was a new search on December 2, 2021 to check whether new studies had been published. All studies were included in the electronic platform Rayyan^©^ QCRI, a web application designed to assist in the selection of articles in systematic reviews.

In PubMed search was made using the following keywords ((((((antibiotic[Title/Abstract]) OR anti-bacterial[Title/Abstract]) OR antimicrobial[Title/Abstract]) OR antibiotic prophylaxis[Title/Abstract]) OR antibiotics[Title/Abstract])) AND (((((fetal membranes[Title/Abstract]) OR premature rupture of membranes[Title/Abstract]) OR premature rupture of fetal membranes[Title/Abstract]) OR preterm premature rupture of fetal membranes[Title/Abstract]) OR PRPM[Title/Abstract]). However, in Cochrane the search was conducted using “antibiotic” OR “anti-bacterial”; OR “antibiotic prophylaxis”; OR “antibiotics”; OR “antimicrobial”; AND “PRPM” OR “premature rupture of membranes”; OR “premature rupture of fetal membranes”; OR “fetal membranes”.

### Eligibility criteria

The evaluated criteria were study design, participants, intervention, and outcomes. We analyzed only randomized clinical trials of pregnant women with PRPM and gestational age between 24 and 37 weeks submitted to antibiotic *versus* placebo. Observational studies, book chapters, commentaries, literature reviews, case reports, experimental studies, or randomized clinical trials including pregnant women with PRPM and who were at gestational age below 24 weeks or above 37 weeks were excluded. Randomized clinical trials without Control Group or Placebo, and/or those that used antibiotic for *Streptococcus agalactiae* were also excluded from analysis.

### Selection of studies

Authors independently assessed all selected studies in the published literature search. When articles written by the same authors were found, their contents were checked in order to establish whether the same sample had been used and, if so, the most complete sample was chosen.

### Data extraction

For data extraction and management, a spreadsheet was created containing the following variables: author and year of article’s publication, gestational age of patients included in the study, number of participants, type of intervention performed, type of Control Group, duration of antibiotic use, use of corticosteroids, magnesium sulfate and tocolytics. We also considered whether the article referred to the treatment of streptococci, gestational age at in which presented ruptured membrane, occurrence of prolonged latency period within days or hours, gestational age at delivery, cesarean delivery, chorioamnionitis, endometritis, maternal sepsis, maternal death, newborn birth weight, fetal death, neonatal death, perinatal death, Apgar score, RDS, NEC, neonatal sepsis, and intraventricular hemorrhage.

### Assessing risk of bias

The risk of bias of studies included in this systematic review and meta-analysis was assessed by the authors in an independent and blind manner as suggested by both PRISMA protocol and Cochrane collaboration.

### Statistical analysis

Meta-analyses were conducted when there were two or more studies that reported the same outcome. The fixed model was used when there was no significant heterogeneity (Cochran test >0.10) and the random model when this heterogeneity was presented. In addition, the Higgns I^2^ test was conducted in order to define whether there was high heterogeneity (> 50%).

Subgroup meta-analyses were performed with the following variables: gestational age up to 34 weeks, the use of Ampicillin alone as an intervention, study blinding, the use of corticosteroids and tocolysis.

Funnel Plot and Deek’s test were performed to assess the risk of publication bias. The data was introduced in Review Manager version 5.3 by the Cochrane Collaboration.

## RESULTS

**Figure 1:**
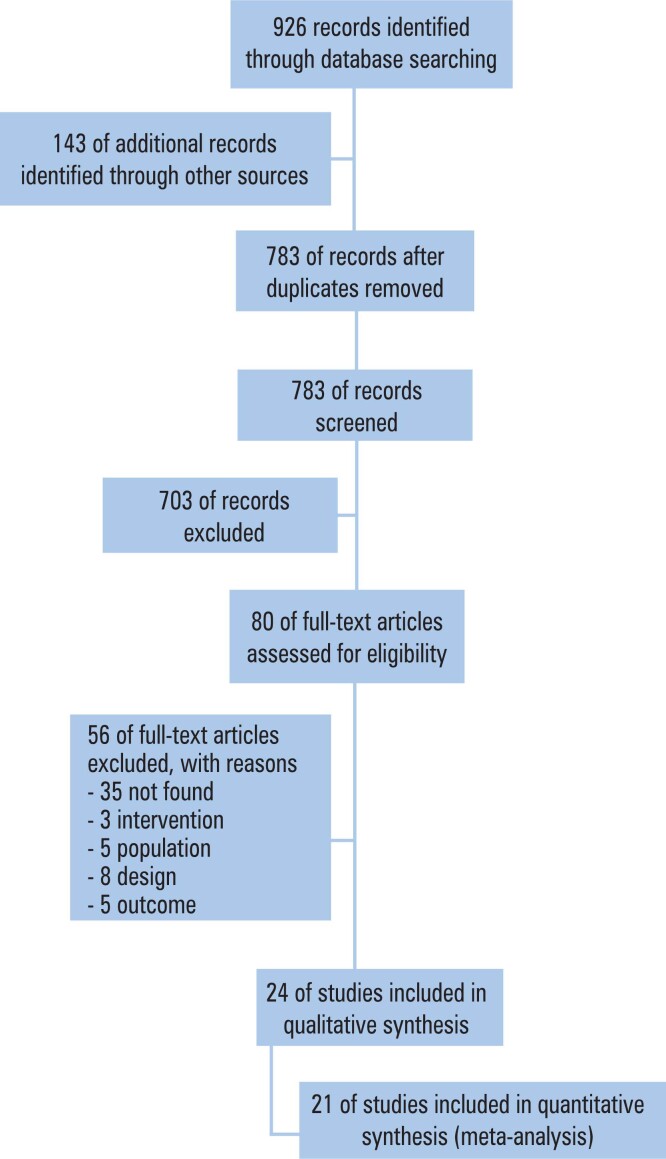
Flowchart showing the steps used during selection of studies included in this systematic review and meta-analysis

A total of 926 manuscripts were retrieved from the search bases. In December 2021 new search, another 112 articles were found. None of the articles submitted to the meta-analysis were the result of second search. In all stages of the study, the articles were read separately by two examiners and disagreements were solved after the discussion with an expert. The flowchart of selection can be seen in figure 1 and main characteristics of included studies are shown in table 1.^(9–29)^ In total, 7,111 pregnant women and their newborns were evaluated.

**Table 1:** Population characteristics of included studies

Author	Gestational age	Number of participants	Gestational age rupture Intervention average/median	Gestational age rupture Control mean/median
Almeida et al.,^(9)^	37	106	32 (±2.1)	31.7 (±4.0)
Amon et al.,^(10)^	34	82	NR	NR
Camli et al.,^(11)^	34	31	NR	NR
Christmas et al.,^(12)^	34	94	30.4	29.9
Cox et al.,^(13)^	34	62	26.9 (±1.9)	26.7 (±2.2)
August Fuhr et al.,^(14)^	34	105	NR	NR
Garcia-Burguillo et al.,^(15)^	37	60	NR	NR
Grable et al.,^(16)^	37	60	NR	NR
Johnston et al.,^(17)^	34	85	29.5 (±0.70)	30.3 (±0.5)
Kenyon et al.,^(18)^	37	4,809	E 27.5 (±6.1) AXC 28.0 (±6.0) AXCE 27.8 (±6.1)	27.9 (±6.1)
Kurki et al.,^(19)^	37	101	32.4 (±2.3)	32.3 (±2.4)
Lockwood et al.,^(20)^	37	75	NR	NR
Lovett et al.,^(21)^	37	102	ASAXC 30.17 (±0.57) AAXC 29.73 (±0.42)	29.58 (±0.54)
Magwali et al.,^(22)^	37	170	32.8 (±2.7)	32.8 (±2.5)
McCaul et al.,^(23)^	34	37	NR	NR
McGregor et al.,^(24)^	37	55	30.5 (±3.5)	31.5 (±2.8)
Mercer et al.,^(25)^	37	220	30.3 (±3.4)	29.8 (±3.6)
Mercer,^(26)^	34	614	28.6 (±2.2)	28.5 (±2.4)
Morales et al.,^(27)^	34	78	29.4 (±2.3)	29.3 (± 2.7)
Ovalle et al.,^(28)^	34	88	29.9 (±2.5)	29.3 (± 2.9)
Svare et al.,^(29)^	34	67	NR	NR

NR: not reported; E: Erythromycin; AXC: Amoxicillin + Clavulanate; AXCE: Amoxicillin + Clavulanate + Erythromycin; ASAXC: Ampicillin + Sulbactam + Amoxicillin + Clavulanate; AAXC: Ampicillin + Amoxicillin + Clavulanate.

The antibiotic regimens varied in the different studies, corresponding to the following therapeutic regimens: A: Ampicillin; AE: Ampicillin + Erythromycin; AM: Ampicillin + Metronidazole; AS: Ampicillin + Sulbactam; AX: Amoxicillin; AXC: Amoxicillin + Clavulanate; AGC: Ampicillin + Gentamycin + Clindamycin; AXCE: Amoxicillin + Clavulanate + Erythromycin; CG: Clindamycin + Gentamycin; E: Erythromycin; MZ: Mezlocillin; MZA: Mezlocillin + Ampicillin; P: Penicillin; PP: Piperacillin; PVM: Pivampicillin + Metronidazole.

Four articles evaluated the use of some antibiotic *versus* another antibiotic, however, due to the absence of a Placebo Control Group, they could not be included in the meta-analysis.^(30–33)^

The duration of treatment was, on average, 7 days, however, some articles mentioned the use of antibiotics until delivery.^(19–29)^ The use of corticosteroids and tocolysis was not adequately reported in some studies, which impaired the assessment. The antibiotic regimen used as intervention, its duration, as well as the presence or absence of corticosteroid therapy or tocolysis, can be analyzed in table 2.

**Table 2:** Characteristics of interventions of the included studies

Author	Intervention	Corticosteroid	Tocolysis	Intervention duration
Almeida et al.,^(9)^	AX 750mg 8/8 hours VO	NR	NR	NR
Amon et al.,^(10)^	A 1g 6/6 hours IV for 24 hours + A 500mg 6/6 hours VO until delivery	SN	Yes	Until birth
Camli et al.,^(11)^	A 1g 6/6 hours IV	NR	No	NR
Christmas et al.,^(12)^	AGC (A 2g 6/6 hours IV + G 60-90mg 8/8 hours IV + C 900mg 8/8 hours IV) 1 day, AXC 500 + 125mg 8/8 hours 7 days VO	No	No	7 days
Cox et al.,^(13)^	AS 1+2g 6/6 hours 4 doses IV, AXC 500 + 125mg 6/6 hours VO for 5 days	NR	NR	5 days
August Fuhr et al.,^(14)^	MZ 2g 8/8 hours IV 7 days	Yes	Yes	7 days
Garcia-Burguillo et al.,^(15)^	E 500mg 6/6 hours VO until birth	No	No	Until birth
Grable et al.,^(16)^	A 2g 6/6 hours IV (1 day) + A 500mg 6/6 hours VO until birth	NR	NR	Until birth
Johnston et al.,^(17)^	MZ por 2 days + A VO	No	No	Until birth
Kenyon et al.,^(18)^	AXC 250 + 125mg 8/8 hours VO + E 250mg VO AXC 250 + 125mg 8/8 hours VO E 250mg	SN	SN	Until birth
Kurki et al.,^(19)^	P 5000mUI 2 doses IV	NR	Yes	1 day
Lockwood et al.,^(20)^	PP 3g 6/6 hours IV 72 hours	SN	SN	3 days
Lovett et al.,^(21)^	AS 1.5g 6/6 hours IV 72 hours + AXC 500+125mg 8/8 hours A 2g IV 72 hours + AXC 500+125mg 8/8 hours	SN	Yes	Until birth
Magwali et al.,^(22)^	AXC VO 5 days	SN	NR	5 days
McCaul et al.,^(23)^	A 2g IV + A 500mg 6/6 hours 7 days	No	No	7 days
McGregor et al.,^(24)^	E 333mg 8/8 hours	No	No	NR
Mercer et al.,^(25)^	E 333mg 8/8 hours	SN	SN	NR
Mercer,^(26)^	A 2g 6/6 hours + E 250mg 6/6 hours IV AX 250mg 8/8 hours VO + E 333mg 8/8 hours VO 5 days	No	No	5 days
Morales et al.,^(27)^	A 2g 6/6 hours	No	No	NR
Ovalle et al.,^(28)^	CG (C 600mg 6/6 hours IV + G 4mg/kg/day 48 hours) and CG (C 300mg 6/6 hours + G 2mg/kg/day 12/12 hours) IM	NR	NR	5 days
Svare et al.,^(29)^	A 2g 6/6 hours IV (1 day) and M 500mg 8/8 hours IV (1 day) + PVM (PV 500mg 8/8 hours 7 days + M 400mg 8/8 hours) VO	NR	NR	7 days

NR: not reported; VO: orally; IV: intravenous; IM: intramuscular; SN: if necessary; AX: Amoxicillin; A: Ampicillin; AGC: Ampicillin + Gentamycin + Clindamycin; AXC: Amoxicillin + Clavulanate; AS: Ampicillin + Sulbactam; MZ: Mezlocillin; E: Erythromycin; P: Penicillin; PP: Piperacillin; CG: Clindamycin + Gentamycin; C: Clindamycin; G: Gentamycin; M: Metronidazole; PV: Pivampicilin; PVM: Pivampicillin + Metronidazole.

The outcomes evaluated were divided into maternal (prolonged latency period, gestational age at delivery, choryamnionitis, endometritis and maternal death), fetal (fetal death) and neonatal (Apgar score, birth weight, RDS, NEC, neonatal sepsis and neonatal death) and they are summarized in tables 3 and 4 and in supplementary table 1.

**Table 3:** Maternal outcome data extracted from included studies

Author	Intervention latency (≥7 days)	Control latency (≥7 days)	Chorioamnionitis Intervention	Chorioamnionitis Control	Endometritis Intervention	Endometritis Control	Maternal sepsis Intervention	Maternal sepsis Control	Maternal death Intervention	Maternal death Control
Almeida et al.,^(9)^	NR	NR	NR	NR	NR	NR	NR	NR	NR	NR
Amon et al.,^(10)^	20/43	10/39	7/43	4/39	5/43	3/39	NR	NR	NR	NR
Camli et al.,^(11)^	NR	NR	NR	NR	NR	NR	NR	NR	NR	NR
Christmas et al.,^(12)^	20/48	7/46	5/48	8/46	3/48	1/46	NR	NR	NR	NR
Cox et al.,^(13)^	NR	NR	NR	NR	NR	NR	NR	NR	NR	NR
August Fuhr et al.,^(14)^	30/47	26/58	NR	NR	NR	NR	NR	NR	NR	NR
Garcia Burguillo et al.,^(15)^	NR	NR	3/30	1/30	NR	NR	NR	NR	NR	NR
Grable et al.,^(16)^	NR	NR	4/31	8/29	NR	NR	NR	NR	NR	NR
Johnston et al.,^(17)^	18/40	8/45	3/40	16/45	5/40	15/45	NR	NR	0/40	0/45
Kenyon et al.,^(18)^	1767/3,584	775/1,225	NR	NR	NR	NR	896/3,584	330/1,225	NR	NR
Kurki et al.,^(19)^	NR	NR	1/50	7/51	0/50	1/51	0/50	0/51	NR	NR
Lockwood et al.,^(20)^	16/38	4/37	10/35	10/37	NR	NR	NR	NR	NR	NR
Lovett et al.,^(21)^	NR	NR	11/75	12/37	NR	NR	NR	NR	NR	NR
Magwali et al.,^(22)^	15/82	6/86	14/82	20/86	NR	NR	10/82	17/86	NR	NR
McCaul et al.,^(23)^	NR	NR	10/41	9/43	NR	NR	10/41	9/43	NR	NR
McGregor et al.,^(24)^	NR	NR	7/28	6/27	NR	NR	NR	NR	NR	NR
Mercer et al.,^(25)^	29/106	20/114	NR	NR	NR	NR	NR	NR	NR	NR
Mercer,^(26)^	NR	NR	69/299	101/312	NR	NR	0/299	0/312	0/299	0/312
Morales et al.,^(27)^	NR	NR	0/37	16/41	NR	NR	NR	NR	NR	NR
Ovalle et al.,^(28)^	NR	NR	2/42	11/45	NR	NR	NR	NR	NR	NR
Svare et al.,^(29)^	NR	NR	6/30	5/37	NR	NR	NR	NR	0/30	0/37

NR: not reported.

**Table 4:** Neonatal outcome data extracted from included studies

Author	Gestational age at delivery Mean intervention (SD)	Gestational age at delivery Mean control (SD)	Weight newborn± Average Intervention (SD)	Weight newborn Control (SD)	Apgar5 <7 Intervention	Apgar5 <7 Control	Neonatal death Intervention	Neonatal death Control
Almeida et al.,^(9)^	NR	NR	2,094 (±459)	2,045 (±462)	NR	NR	NR	NR
Amon et al.,^(10)^	31.5 (±3.3)	30.7 (±3.2)	1,670 (±580)	1,742 (±846)	5/42	6/36	1/42	3/36
Camli et al.,^(11)^	NR	NR	NR	NR	NR	NR	NR	NR
Christmas et al.,^(12)^	31.4	30.7	1,639	1,839	NR	NR	NR	NR
Cox et al.,^(13)^	26.8 (±5.4)	27.9 (±2.1)	1,282 (±409)	1,305 (±413)	NR	NR	1/31	5/31
August Fuhr et al.,^(14)^	NR	NR	NR		NR	NR	NR	NR
Garcia-Burguillo et al.,^(15)^	NR	NR	2,022 (±607)	2,170 (±799.7)	NR	NR	NR	NR
Grable et al.,^(16)^	NR	NR	NR	NR	NR	NR	NR	NR
Johnston et al.,^(17)^	NR	NR	1,897 (±600)	1,587 (±592)	NR	NR	3/40	3/45
Kenyon et al.,^(18)^	NR	NR	2,103 (±764)	2,072 (±769)	NR	NR	226/3,584	82/1,225
Kurki et al.,^(19)^	33.2 (±2.1)	33.1 (±1.8)	2,124 (±390)	2,090 (±516)	5/57	8/58	1/57	1/58
Lockwood et al.,^(20)^	31.7 (±3.5)	31.2 (±3.3)	1,837 (±759)	1,697 (±581)	3/37	3/35	2/37	2/35
Lovett et al.,^(21)^	NR	NR	ASAXC 1,870 (±101) AAXC 1,674 (±71)	1,543 (±95)	NR	NR	0/76	3/37
Magwali et al.,^(22)^	NR	NR	1,985	2,150	NR	NR	8/82	11/86
McCaul et al.,^(23)^	30.1 (±2.7)	31.2 (±3.9)	1,724 (±587.3)	1,925.3 (±842.5)	NR	NR	NR	NR
McGregor et al.,^(24)^	31.4 (±3.4)	32.3 (±2.3)	1,638,5 (±530,8)	1,741,4 (±444)	NR	NR	6/28	0/27
Mercer et al.,^(25)^	NR	NR	1,771 (±653)	1,817 (±637)	15/107	20/109	1/106	3/114
Mercer,^(26)^	NR	NR	1,549 (±497)	1,457 (±508)	NR	NR	19/299	18/312
Morales et al.,^(27)^	NR	NR	NR	NR	NR	NR	NR	NR
Ovalle et al.,^(28)^	NR	NR	1,849 (±458.4)	1,645 (±521.4)	NR	NR	4/11	3/13
Svare et al.,^(29)^	NR	NR	1,962 (±712)	1,838 (±785)	NR	NR	NR	NR

NR: not reported; SD: standard deviation; ASAXC: Ampicillin + Sulbactam + Amoxicillin + Clavulanate; AAXC: Ampicillin + Amoxicillin + Clavulanate.

### Extension of the latency period

**Figure 2:**
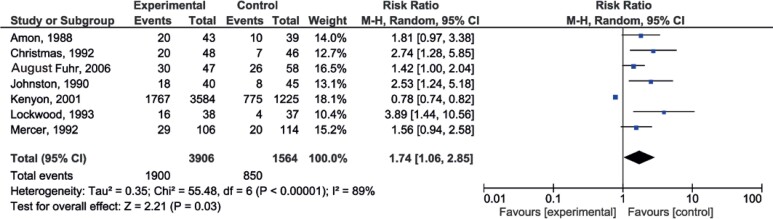
Forest plot of latency time ≥ 7 days

Studies were standardized in units of hours or days. The analysis showed no difference between the antibiotic and Placebo Groups (p=0.25). However, when categorized in a period greater than or equal to 7 days, there was an increase in latency time in the Antibiotic Group (p=0.03), and relative risk (RR) of 1.74 (1.06-2.85) as it can be seen in figure 2.

**Supplementary figure 2.1:**
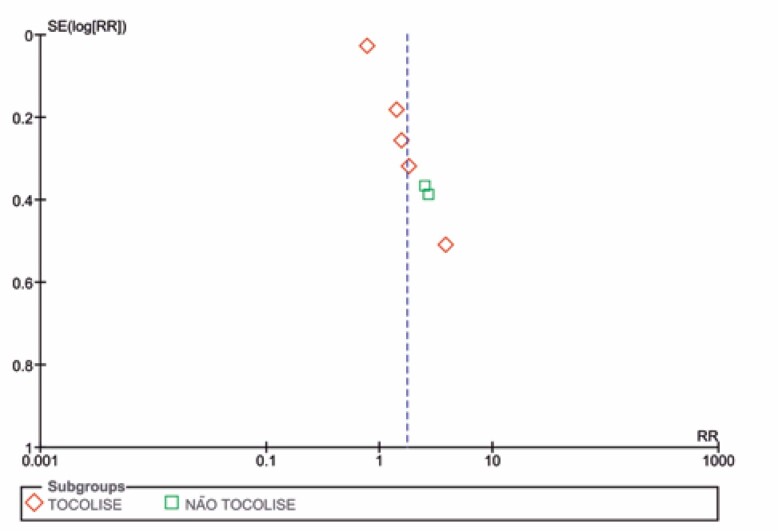
Funnel plot of latency time ≥7 days

Regarding the latency period greater than 7 days, the funnel plot did not present adequate distribution (Supplementary figure 2.1).

### Prolongation of the latency period with corticosteroids

The absence of corticosteroids while using antibiotic prophylaxis was correlated with an increase in the latency period in the group categorized in days.

### Gestational age at birth

**Supplementary figure 2.2:**
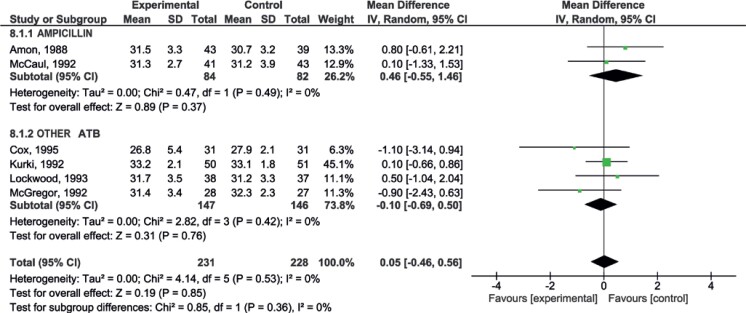
Forest plot of gestational age at delivery

There was no statistically significant difference in terms of gestational age at delivery between the Placebo Group and the group that used antibiotics (p=0.85). This outcome was analyzed in only six studies due to the lack of data in the other manuscripts (Supplementary figure 2.2).

### Chorioamnionitis

**Figure 3:**
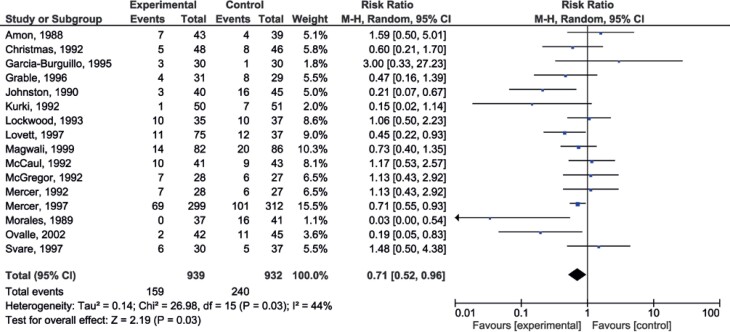
Forest plot of chorioamnionitis

The use of antibiotics demonstrated protection against chorioamnionitis (p=0.03) with RR=0.71 (0.52-0.96) (Figure 3). When chorioamnionitis was evaluated along with gestational age, protection was statistically more significant in the group of pregnant women with gestational age up to 34 weeks.

Five studies used ampicillin alone. The comparison between the ampicillin Group Alone *versus* the Group of other Antibiotics showed no statistical difference in relation to chorioamnionitis.

**Supplementary figure 3.1:**
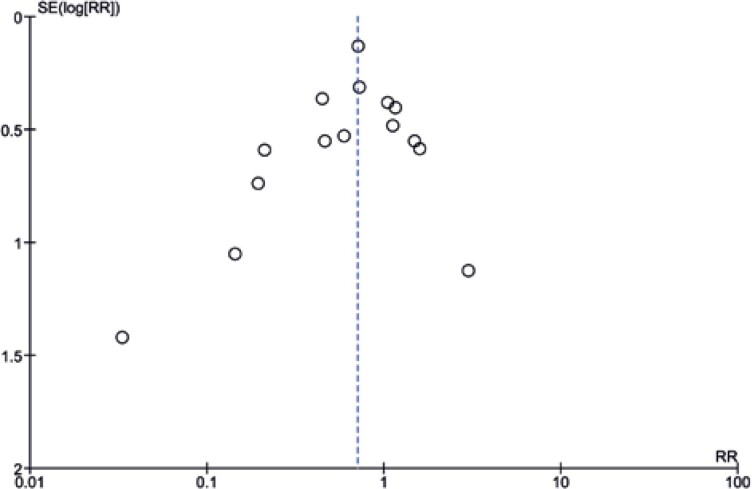
Chorioamnionitis funnel-plot

The funnel plot showed an adequate distribution of studies (Supplementary figure 3.1).

### Endometritis

**Supplementary figure 3.2:**
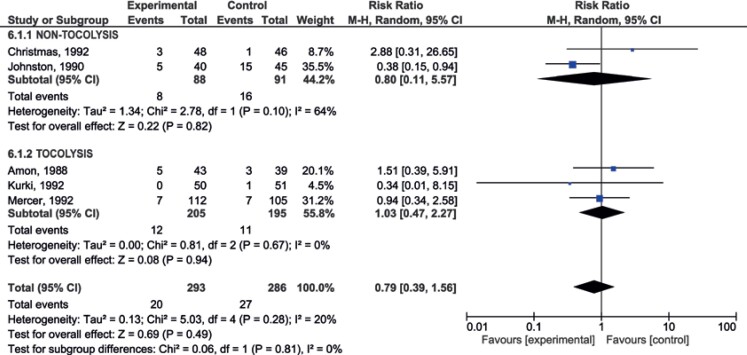
Forest plot of endometritis

There was no statistically significant difference between the Group that used Antibiotics *versus* the Placebo Group (p=0.49 and 95%CI: 0.39–1.56) (Supplementary figure 3.2).

### Maternal death

There were no records of maternal deaths in the meta-analyzed studies.

### Apgar score < 7 in the fifth minute

**Supplementary figure 3.3:**
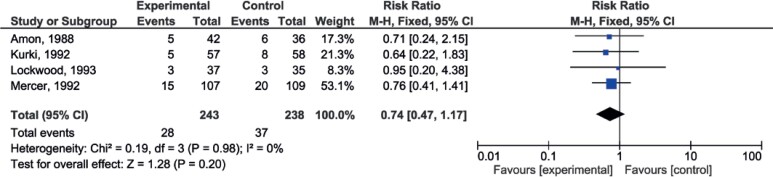
Forest plot of Apgar < 7 fifth minute

There was no statistically significant difference between the Groups that used Antibiotics *versus* Placebo (RR=0.74 and 95%CI: 0.47-1.17) supplementary figure 3.3.

### Birth weight

**Supplementary figure 3.4:**
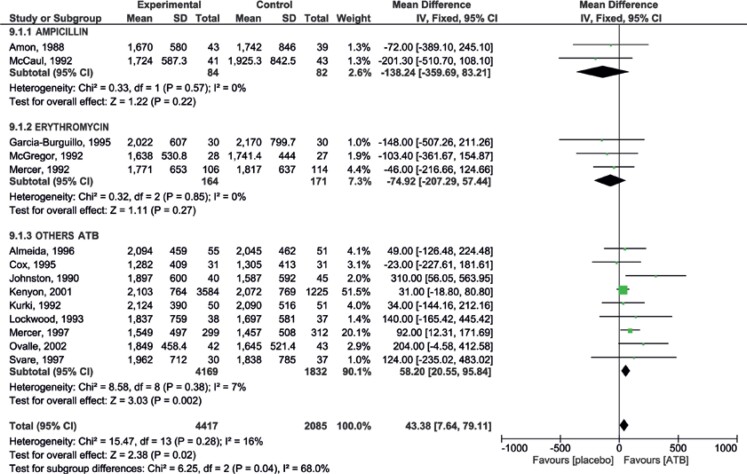
Forest plot of birth weight

In the Group in which the pregnant women used Antibiotics, the newborns presented, on average, 43.38g more than in the Placebo Group (p=0.02) with a confidence interval ranging from 7.64 to 79.11g. The funnel plot showed an adequate distribution (Supplementary figure 3.4).

### Respiratory distress syndrome

**Supplementary figure 3.5:**
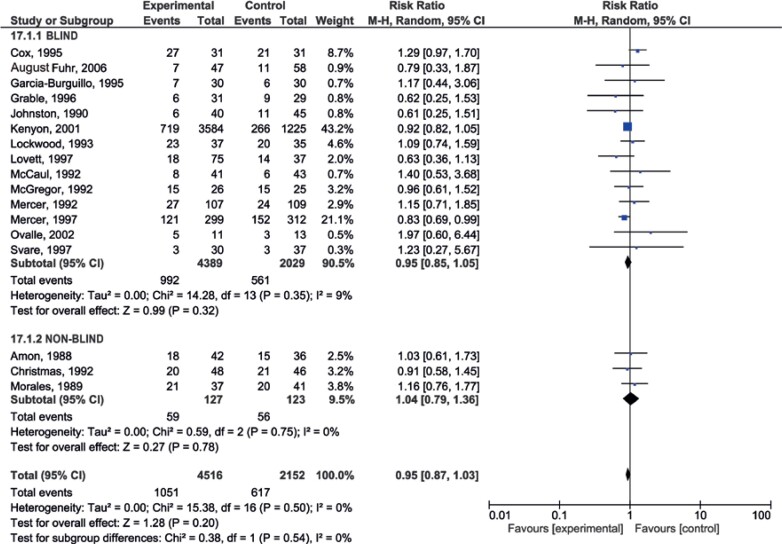
Forest plot of respiratory distress syndrome

There was no statistically significant difference between the Groups that used Antibiotics *versus* Placebo (RR=0.95 and 95%CI: 0.87-1.03) supplementary figure 3.5.

### Necrotizing enterocolitis

**Supplementary figure 3.6:**
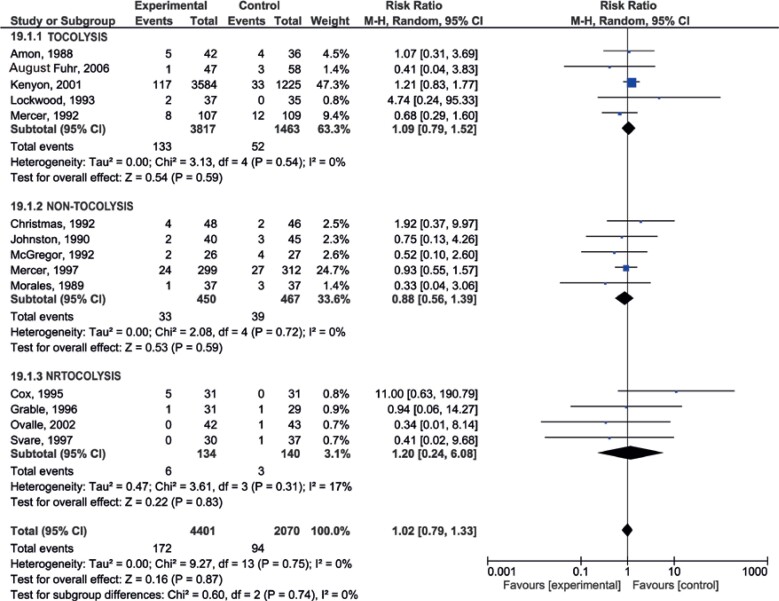
Forest plot of necrotizing enterocolitis

There was no statistically significant difference between the Groups that used Antibiotics *versus* Placebo (RR=1.02 and 95%CI: 0.79-1.33) supplementary figure 3.6.

### Neonatal sepsis

The use of antibiotics reduces the risk of neonatal sepsis, with a confidence interval between 0.69 and 0.88, therefore, revealing a protective effect of 22%. The effect of antibiotic use was similar in studies that evaluated gestational age up to 34 weeks and in those that evaluated up to 37 weeks.

When comparing the use of ampicillin *versus* erythromycin *versus* other antibiotics, the protective effect was not revealed in the erythromycin subgroup.

**Supplementary figure 3.7:**
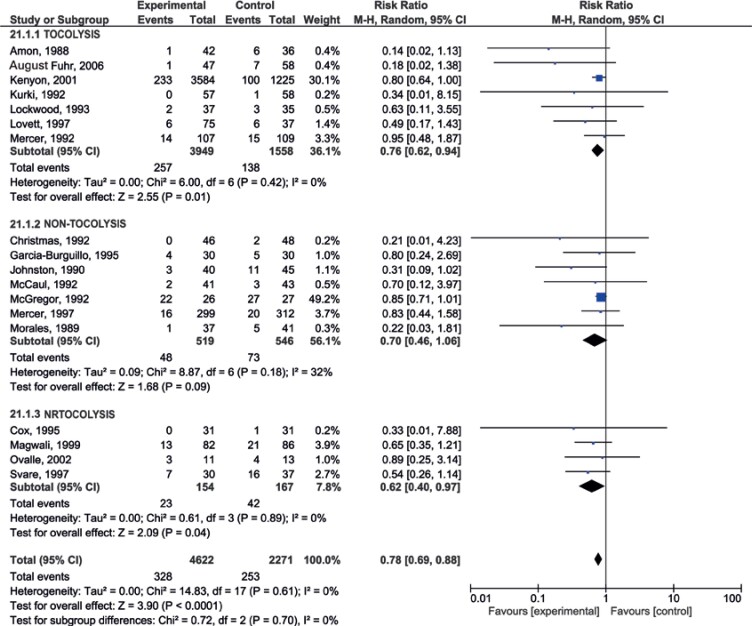
Forest plot of neonatal sepsis

The studies did not clarify the period of occurrence of neonatal sepsis, that is, whether early or late supplementary figure 3.7.

### Neonatal and perinatal death

**Supplementary figure 3.8:**
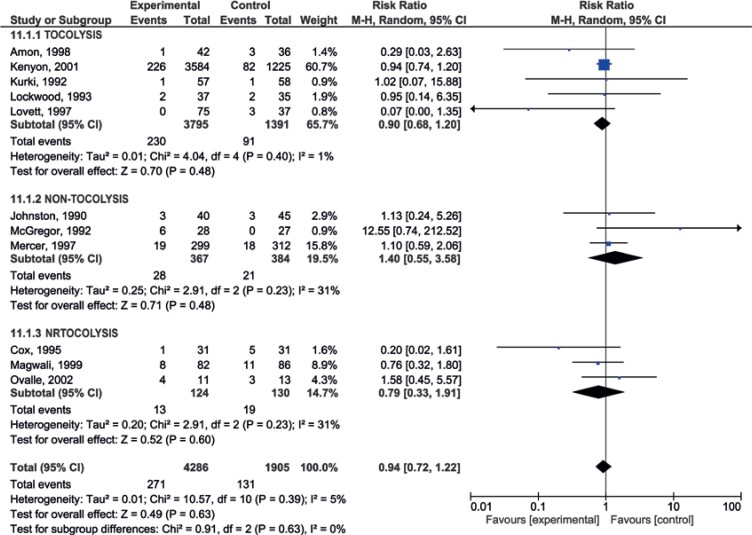
Forest plot of neonatal death

**Supplementary figure 3.9:**
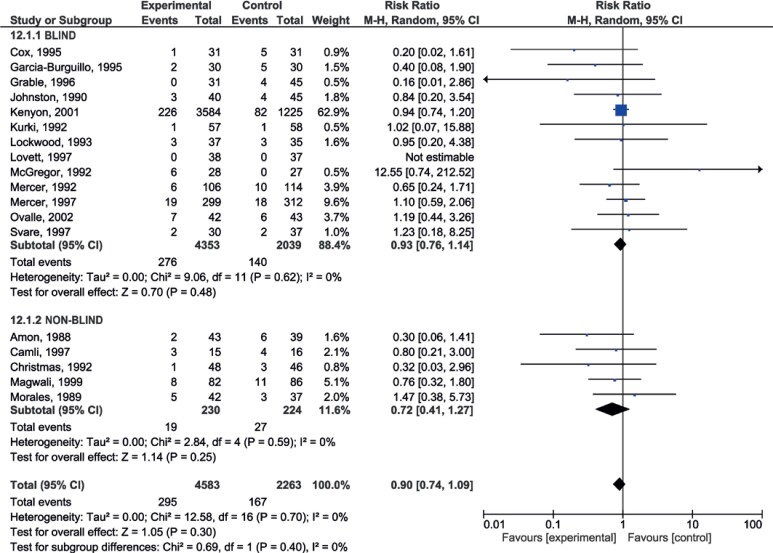
Forest plot of perinatal death

There was no statistically significant difference between the Groups of pregnant women who used Antibiotics *versus* Placebo (RR=0.94 and 95%CI: 0.72-1.22) supplementary figures 3.8 and 3.9.

### Fetal death

**Supplementary figure 3.10:**
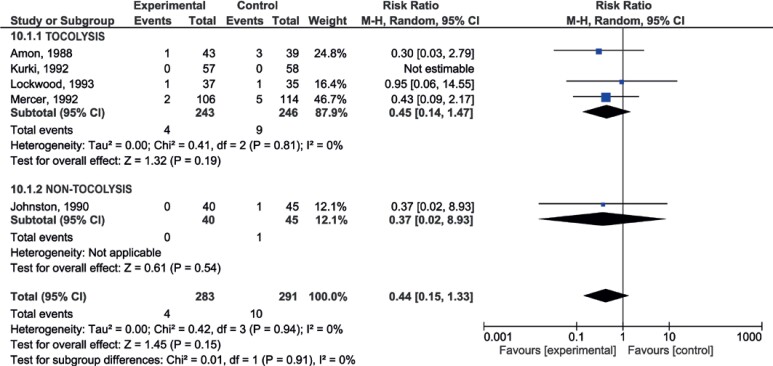
Forest plot of fetal death

There was no statistically significant difference between the Groups of pregnant women who used Antibiotics *versus* Placebo (p=0.44 and 95%CI: 0.15-1.33) supplementary figure 3.10.

## DISCUSSION

This review was prepared from randomized clinical trials, which are studies with a high degree of evidence. The first, and perhaps most important finding of this study is the publication date of the last randomized clinical trial on the subject. The most recent article found, after a comprehensive search, was published in 2013.^(30)^ Given this observation, some hypotheses can be proposed: the first would be that the medical community had lost interest in the subject, which does not seem to express the reality, since that there are numerous more recent retrospective studies on the subject.^(34–37)^ Another explanation for such questioning would be that the interventions necessary to carry out clinical trials began to face ethical questions related to the use of antibiotics, since, if the use of antibiotics is beneficial in these situations, it would be unethical and iatrogenic to make a study with a Placebo Control Group. It goes without saying that some clinical trials are sponsored by pharmaceutical companies, that prohibit the publication of negative results which can also explain the lack of studies.

Regarding the selection of articles, it is pertinent to comment that only those referring to the use of broad-spectrum antibiotic therapy that increased the latency period were included in this study, as well as those that mentioned the use of antibiotics for the exclusive purpose of preventing neonatal sepsis due to streptococcal disease. Such an action may change the actual results of the study, since some studies showed the role of *Streptococcus agalactiae* as a cause of prematurity and PRPM.^(37–39)^

The use of antibiotics seems to prolong the latency period in pregnant women with premature rupture of preterm membranes. This is a protective factor against chorioamnionitis and neonatal sepsis, in addition this seems to be beneficial for newborn weight gaining, as proposed by several authors.^(30–32,34)^ Such observation, at first, may suggest that the use of antibiotics is valid and should be incorporated into health service protocols. However, this finding should be analyzed with caution since the present study has limitations, such as lack of recent clinical trials and inconsistency regarding the dosage and antibiotic regimen.

The articles that analyzed the use of Ampicillin alone did not show statistically significant differences compared with studies that used other antibiotic prophylaxis regimens to prevent chorioamnionitis. Such finding should lead to the questioning about the real need for broad-spectrum antibiotic therapy to prevent this outcome, or whether just the use of antibiotic prophylaxis with Ampicillin is imperative.^(9–10,22)^

Regarding the latency period due to the heterogeneity of data reporting in the different studies, it was only possible to observe the statistical difference when the latency period was categorized as < or ≥7 days. When evaluating the period of ≥7 days, a benefit was observed with the use of antibiotics. One possibility, suggested by the funnel plot, to explain different benefits in two similar groups, would be the occurrence of publication bias, which is the tendency of authors or journals to publish positive or more significant evidence, even when they are identified in studies with limited scientific evidence.

Another controversial finding was that antibiotic prophylaxis was beneficial in preventing chorioamnionitis, with no evidence of preventing endometritis. This finding contradicted the authors’ hypotheses, who expected that the use would protect against all infectious outcomes, as described in other studies.

Unfortunately, important neonatal outcomes were not verified because there were no statistically significant differences between the Group that used Antibiotics and the Control Group, including: NEC, RDS and neonatal death.^(40–42)^ If the studies had shown statistical significance in any of these variables, they could have contributed to conclusive results on the beneficial effect of antibiotics.

In the present study, the antibiotic regimens evaluated varied and it was not possible to define the most appropriate one. In addition, some of the antibiotics used, such as erythromycin orally, are not available in the Brazilian pharmaceutical market. Thus, given the lack of consistent results in the literature, many services continue to use Ampicillin 2g intravenously every 6 hours for 48 hours, followed by amoxicillin 500mg 8/8h for 5 days, and azithromycin 1g orally, a regimen that covers *Streptococcus agalactiae*, Gram negative (*Neisseria gonorrhoeae, Escherichia coli*) and atypical bacteria such as *Chlamydia* e *Ureaplasma*, present in the vaginal flora.^(39,42)^

Most protocols for the management of pregnancy with premature rupture of preterm membranes recommend interruption after 34 weeks.^(43,44)^ However, after reading several articles, it was evidenced that, in some studies, there is a tendency to keep the pregnancy until term with the use of antibiotic prophylaxis. Therefore, to define the ideal moment for the resolution of the pregnancy, further studies are needed to assess the risks and benefits of maintaining these pregnancies up to 37 weeks.

A major limitation of the study is related to the heterogeneity (very serious or serious concern during analysis and I² higher than 50%) and diversity of interventions found in the articles studied, which prevented the presence of more robust results, which did not allow the conclusion of the best antibiotic therapy regimen, since several different regimens were studied.

Finally, this review can benefit in the assembly of a possible guideline or protocol on the subject, given that the vast majority of published studies in this area failed to reach a consensus. As the study showed a reduction in chorioamnionitis, neonatal sepsis and an increased latency period, the use of antibiotic therapy in pregnant women with PRPM should always be considered.

## CONCLUSION

This study showed that the use of antibiotic prophylaxis in pregnant women with premature rupture of preterm membranes is beneficial for the following variables, such as increased latency period, higher weight at birth, protective factor against chorioamnionitis, as well as neonatal sepsis. There was no statistically significant difference from the other outcomes.

After the evaluation of numerous studies, associated with the results of this review, which presented controversial outcomes, it can be concluded that new randomized clinical trials are needed to ensure the beneficial effect of antibiotic prophylaxis in prolonging the latency period.

## Appendix

**Supplementary table 1:** Neonatal comorbidity data extracted from included studies

Author	RDS Intervention	RDS Control	NEC Intervention	NEC Control	Neonatal sepsis Intervention	Neonatal sepsis Control
Almeida et al.,^(9)^	NR	NR	NR	NR	NR	NR
Amon et al.,^(10)^	18/42	15/36	5/42	4/36	1/42	6/36
Camli et al.,^(11)^	NR	NR	NR	NR	NR	NR
Christmas et al.,^(12)^	20/48	21/46	4/48	2/46	0/46	2/48
Cox et al.,^(13)^	27/31	21/31	5/31	0/31	0/31	1/31
August Fuhr et al.,^(14)^	7/47	11/58	1/47	3/58	1/47	7/58
Garcia-Burguillo et al.,^(15)^	7/30	6/30	NR	NR	4/30	5/30
Grable et al.,^(16)^	6/31	9/29	1/31	1/29	NR	NR
Johnston et al.,^(17)^	6/40	11/45	2/40	3/45	3/40	11/45
Kenyon et al.,^(18)^	719/3,584	266/1,225	117/3,584	33/1,225	233/3,584	100/1,225
Kurki et al.,^(19)^	NR	NR	NR	NR	0/57	1/58
Lockwood et al.,^(20)^	23/37	20/35	2/37	0/35	2/37	3/35
Lovett et al.,^(21)^	18/75	14/37	NR	NR	6/75	6/37
Magwali et al.,^(22)^	NR	NR	NR	NR	13/82	21/86
McCaul et al.,^(23)^	8/41	6/43	NR	NR	2/41	3/43
McGregor et al.,^(24)^	15/26	15/25	2/26	4/27	1/24	1/27
Mercer et al.,^(25)^	27/107	24/109	8/107	12/109	14/107	15/109
Mercer,^(26)^	121/299	152/312	24/299	27/312	16/299	20/312
Morales et al.,^(27)^	21/37	20/41	1/37	3/37	1/37	5/41
Ovalle et al.,^(28)^	5/11	3/13	0/42	1/43	7/42	7/43
Svare et al.,^(29)^	3/30	3/37	0/30	1/37	7/30	16/37

RDS: respiratory distress syndrome; NEC: necrotizing enterocolitis; NR: not reported.
